# Aspiration Thrombectomy in Patients with Acute Myocardial Infarction—5-Year Analysis Based on a Large National Registry (ORPKI)

**DOI:** 10.3390/jcm9113610

**Published:** 2020-11-09

**Authors:** Rafał Januszek, Zbigniew Siudak, Krzysztof P. Malinowski, Roman Wojdyła, Piotr Mika, Wojciech Wańha, Tomasz Kameczura, Andrzej Surdacki, Wojciech Wojakowski, Jacek Legutko, Stanisław Bartuś

**Affiliations:** 1 2nd Department of Cardiology and Cardiovascular Interventions, University Hospital, 30-688 Kraków, Poland; romanwojdyla@gmail.com (R.W.); andrzej.surdacki@uj.edu.pl (A.S.); stanislaw.bartus@uj.edu.pl (S.B.); 2 Department of Clinical Rehabilitation, University of Physical Education, 31-571 Kraków, Poland; piotrmika@poczta.fm; 3 Collegium Medicum, Jan Kochanowski University, 25-317 Kielce, Poland; zbigniew.siudak@gmail.com; 4 2nd Department of Cardiology, Institute of Cardiology, Jagiellonian University Medical College, 31-008 Kraków, Poland; krzysztof.piotr.malinowski@gmail.com; 5 Department of Cardiology and Structural Heart Diseases, Medical University of Silesia, 40-635 Katowice, Poland; wojciech.wanha@gmail.com (W.W.); wwojakowski@sum.edu.pl (W.W.); 6 Chair of Electroradiology, Faculty of Medicine, University of Rzeszow, 35-310 Rzeszow, Poland; tomasz_kameczura@yahoo.com; 7 Department of Interventional Cardiology, Institute of Cardiology, Jagiellonian University Medical College, The John Paul II Hospital, 31-202 Kraków, Poland; jacek.legutko@uj.edu.pl

**Keywords:** acute myocardial infarction, no-reflow phenomenon, primary percutaneous coronary intervention, thrombus aspiration

## Abstract

Blood flow restoration after primary percutaneous coronary intervention (pPCI) in patients with acute myocardial infarction (AMI) may not always be achieved and could be complicated by the no-reflow phenomenon (NRP). The aim of the current study was to assess the frequency of thrombus aspirations (TAs) and NRPs in patients with AMI and treated with pPCI based on the data collected during a 5-year period in the national ORPKI registry, as well as the frequency of periprocedural strokes and predictors of TA and NRP. This retrospective analysis was performed on prospectively collected data gathered in the Polish National Registry of Percutaneous Coronary Interventions (ORPKI), which covered the period between January 2014 and December 2018, and included 200,991 patients treated due to AMI out of 535,857 patients treated using PCI. Among them, 16,777 patients underwent TA. TA was mainly used in the STEMI subgroup of 14,207 patients (84.8%). The frequency of NRP among AMI patients in the thrombectomy group was 2.75% and in the non-thrombectomy group 0.82%. Predictors of TA and NRP were also assessed using multivariate analysis. The percentage of patients treated with pPCI and with PCI alone increased significantly in all of the three selected groups of patients from 88.7% to 94.3% in the AMI group (*p* < 0.001), from 82.3% to 90.3% in the STEMI subgroup (*p* < 0.001), and from 96.3% to 98.2% in the NSTEMI subgroup (*p* < 0.001) during the analysed period. NRP occurred more often in the thrombectomy group for the NSTEMI (0.58% vs. 3.07%, *p* < 0.05) and STEMI (1.06% vs. 2.69%, *p* < 0.05) subgroups. Periprocedural stroke occurred more often in the thrombectomy group in comparison to the non-thrombectomy group with AMI (0.03% vs. 0.01%, *p* < 0.05) and the NSTEMI (0.16% vs. 0.02%, *p* < 0.05). In conclusion, the frequency of TA has been experiencing a steady decline in recent years, regardless of AMI type, among patients treated with pPCI.

## 1. Introduction

Despite the ability of primary percutaneous coronary interventions (pPCIs) to restore patency of infarct-related arteries (IRAs), satisfactory myocardial reperfusion may not always be achieved in patients with a high thrombus burden [1]. Distal embolisation during PCI may result in microvascular obstruction and impaired myocardial blush, leading to a large infarct size (IS) and poorer long-term prognosis [2]. The results of the TAPAS trial (Thrombus Aspiration during PCI in Acute Myocardial Infarction Study) allowed the report that thrombus aspiration (TA) decreased all-cause and cardiovascular mortality rates at 1 year, and tended to reduce the risk of stent thrombosis (ST) compared to PCI alone [3]. However, in two larger randomised control trials, the TASTE (Thrombus Aspiration in STEMI (ST-segment elevation myocardial infarction) in Scandinavia) and TOTAL (Trial of Routine Aspiration Thrombectomy with PCI versus PCI-alone in Patients with STEMI) studies, no benefits of TA were found in reducing the risk of all-cause or cardiovascular mortality, recurrent MI, or ST [4,5]. In the TOTAL study, TA was found to be associated with increased stroke rates at 30 days and 1 year [4]. In contrast to the randomised control trial (RCT), the real-world use of TA is more selective, and is based on the physician’s judgment of thrombus burden, coronary flow, and specific anatomy of the target lesions. The prognosis of pPCI has been demonstrated to be associated with hospital and operator volumes [6]. PPCI is crucial in treatment because it is associated with a significant reduction in morbidity and mortality by the restoration of epicardial blood flow. However, there is a risk of distal embolisation and microvascular occlusion after stent placement or balloon dilatation, which is called the no-reflow phenomenon (NRP). Actual guidelines have demonstrated the role of TA during pPCI by giving class III to routine TA in STEMI patients instead of an IIb recommendation for the use of selective or bail-out TA in previous guidelines [7]. While treatment with TA has not been mentioned in the previous non-ST-segment elevation myocardial infarction (NSTEMI) guidelines, routine TA has not been proven beneficial in this setting and in the actual one is not advised routinely [8,9].

The aim of the current study was to assess the frequency of TAs and NRPs in patients with AMI and treated with pPCIs based on the data gathered during a 5-year period in the national ORPKI registry, as well as the frequency of periprocedural strokes and predictors of TA and NRP.

## 2. Methods

### 2.1. Study Design and Patient Population

This retrospective analysis was performed on prospectively collected data. Data for conducting the current study were obtained from the Polish National Registry of Percutaneous Coronary Interventions (ORPKI) [10]. Data were collected between January 2014 and December 2018. We selected 200,991 patients treated due to AMI out of 535,857 treated using PCI during the analysed period. Among them, 16,777 patients underwent TA. TA was mainly used in the STEMI subgroup of 14,207 patients (84.8%). Technical aspects of the procedure, such as the choice of access site (femoral or radial sheath), catheter size, as well as guidewires, type of thrombectomies, and other devices, were at the operator’s discretion. The decision on TA usage was also left to the operator’s decision. Patients with AMI were qualified for PCI according to current European Guidelines [7,8]. Furthermore, periprocedural anticoagulation and indications for PCI, as well as stent type, also remained at the first operator’s discretion. Antiplatelet therapy was implemented according to current European Guidelines [11]. The protocol complied with the 1964 Declaration of Helsinki, and all participants provided their written informed consent for the percutaneous intervention. Due to the retrospective nature as well as anonymisation of the collected data and registry, obtaining the consent of the Bioethics Committee was not required.

### 2.2. Endpoints

The primary aim of this study was to assess the frequency of TA according to the type of MI in patients treated with pPCI as well as NRP and stroke. The secondary study endpoints included the assessment of possible predictors of TA use and NRP occurrence.

### 2.3. Statistical Analysis

Categorical variables are presented as numbers and percentages. Continuous variables are expressed as mean (standard deviation, (SD)) or median (interquartile range, (IQR)), where applicable. Normality was assessed via the Shapiro–Wilk test. Equality of variance was evaluated using Levene’s test. Differences between the two groups were compared using the Student’s or Welch’s *t*-test, depending on the equality of variances for normally distributed variables. The Mann–Whitney U test was applied for non-normally distributed continuous variables. Categorical variables were compared with Pearson’s chi-squared or Fisher’s exact test if 20% of cells had an expected count of less than 5 (Monte Carlo simulation for Fisher’s test using tables of higher dimensions than 2 × 2). Multifactorial logistic regression models were constructed to find predictors of TA. The best model was obtained using the stepwise regression with minimisation of the Bayesian information criterion as target. Statistical analysis was performed using the JMP, version 15.2.0 (SAS Institute Inc., Cary, NC, USA, 2020).

## 3. Results

### 3.1. Frequency of TA

**Figure 1:**
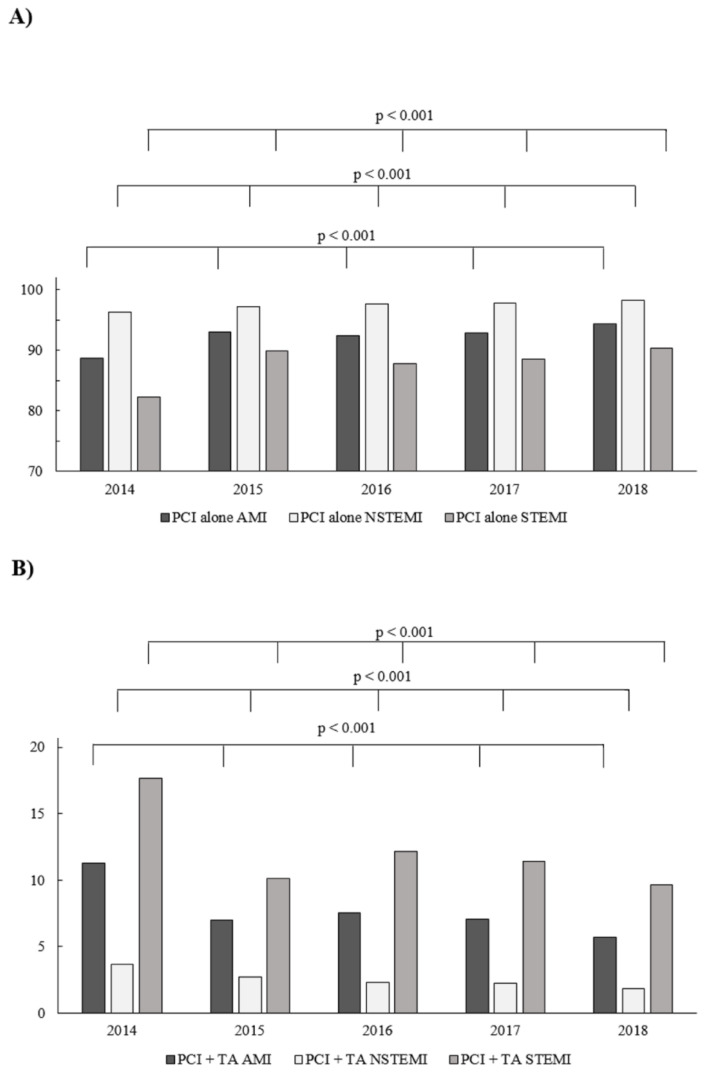
(**A**) The percentage of non-thrombectomy percutaneous coronary interventions (PCIs) according to type of acute myocardial infarction (AMI) in consecutive years (2014–2018) among patients treated with pPCI. (**B**) The frequency of thrombus aspiration (TA) according to type of AMI in consecutive years (2014–2018) among patients treated with primary PCI (pPCI).

Considering the period between 2014 and 2018, the percentage of patients treated with pPCIs, without the use of TA, significantly increased in all of the three selected groups of patients, from 88.7% to 94.3% in the overall AMI group (*p* < 0.001), from 82.3% to 90.3% in STEMI subgroup (*p* < 0.001), and from 96.3% to 98.2% in NSTEMI subgroup (*p* < 0.001; Figure 1A). Furthermore, there were proportionally significant decreases in the frequency of TA usage in the overall group of patients with AMI (*p* < 0.001), as well as in the STEMI (*p* < 0.001) and NSTEMI (*p* < 0.001) subgroup (Figure 1B). Post hoc analysis comparing the significance of differences between individual years in the PCI-TA group is presented in Table 1.

### 3.2. Periprocedural Complications according to Thrombectomy Status

The frequency of NRP in the thrombectomy group was 2.75% in the overall AMI group, while in the non-thrombectomy group, it totalled 0.82% (*p* < 0.05). NRP also occurred more frequently in the thrombectomy group when compared to the non-thrombectomy group in the NSTEMI (0.58% vs. 3.07%, *p* < 0.05) and STEMI (1.06% vs. 2.69%, *p* < 0.05) subgroups. Procedure-related stroke occurred more often in the overall group of patients with AMI in the thrombectomy group compared to the non-thrombectomy group (0.03% vs. 0.01%, *p* < 0.05), and in the NSTEMI subgroup (0.16% vs. 0.02%, *p* < 0.05), while there was no significant difference for STEMI. Those and other selected procedure-related complications as well as relationship between type of MI, occurrence of NRP and direct transport are presented in Table 2 and Table 3. 

### 3.3. Type of PCI according to Thrombectomy Status

Comparing the overall group of patients with AMI, the frequency of failed stent implantation was lower in the thrombectomy group when compared to the non-thrombectomy group (9.8% vs. 8.85%, *p* < 0.05). The frequency of TA use in the NSTEMI group in 2014 (before guidelines change) was 3.65% and between 2015 and 2018 (after guidelines change) it was 2.28%, which was significantly different (*p* < 0.001). Additionally, in the STEMI group, the frequency of TA use in 2014 was 17.7%, and it dropped to 10.8% in the period between 2015 and 2018, which was also statistically significant (*p* < 0.001). These and other indices regarding the type of PCI according to TA status and type of MI are presented in Table 3.

### 3.4. Predictors of No-Reflow and AT in the AMI Group

**Figure 2:**
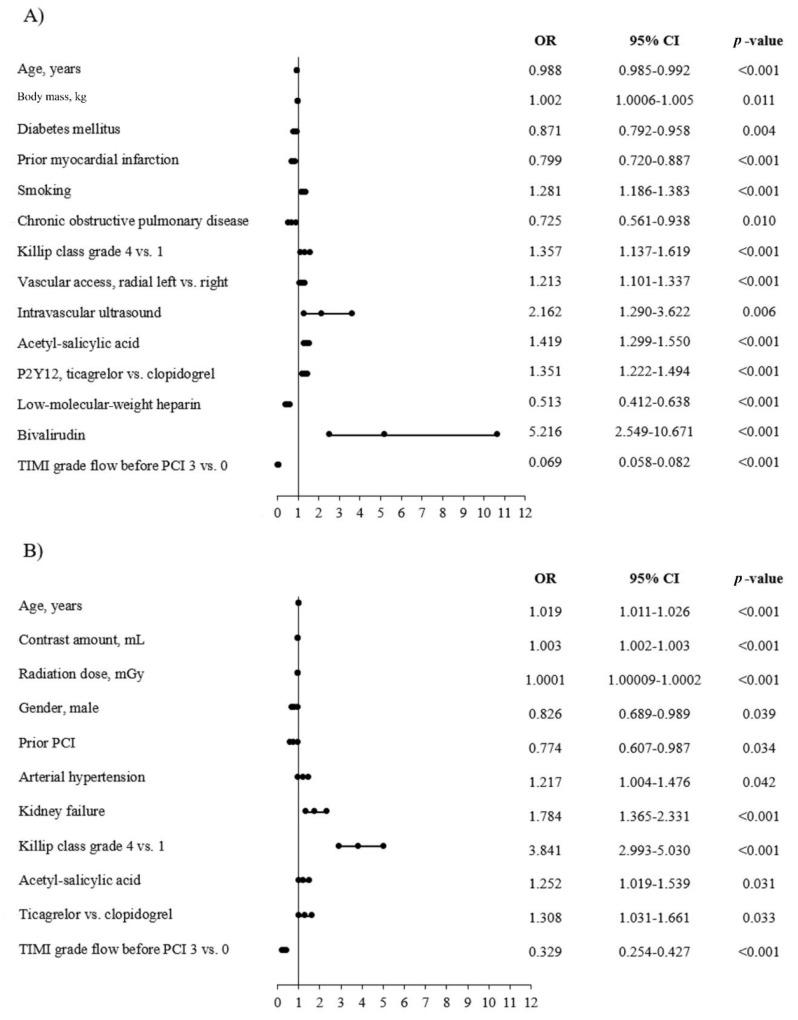
Predictors of TA and no-reflow phenomenon in the overall group of patients with AMI. (**A**) Thrombus aspiration. (**B**) No-reflow.

The results of univariate analysis are presented in Table 4. Among predictors of increased TA usage rate, we found greater body mass, smoking, higher grade of Killip class at admission, the use of intravascular ultrasound, treatment with acetyl-salicylic acid before PCI, treatment with new P2Y_12_ inhibitors (ticagrelor vs. clopidogrel), vascular access, and treatment with bivalirudin. Among predictors of lower frequency of TA use, we found older age, diabetes, prior MI, COPD, treatment with low-molecular-weight heparin (LMWH) before PCI, and patency of culprit artery before PCI. Among predictors of higher frequency of NRP, we demonstrated older age, contrast amount, radiation dose, arterial hypertension, kidney failure, higher grade of Killip class at admission, treatment with acetyl-salicylic acid (ASA), and with new P2Y_12_ inhibitors (ticagrelor vs. clopidogrel). Whereas, among predictors of lower NRP frequency, we found male gender, prior PCI, and patent coronary artery before PCI. The results of univariate analysis are presented in Figure 2A,B.

### 3.5. Predictors of No-Reflow and AT in the STEMI Group

**Figure 3:**
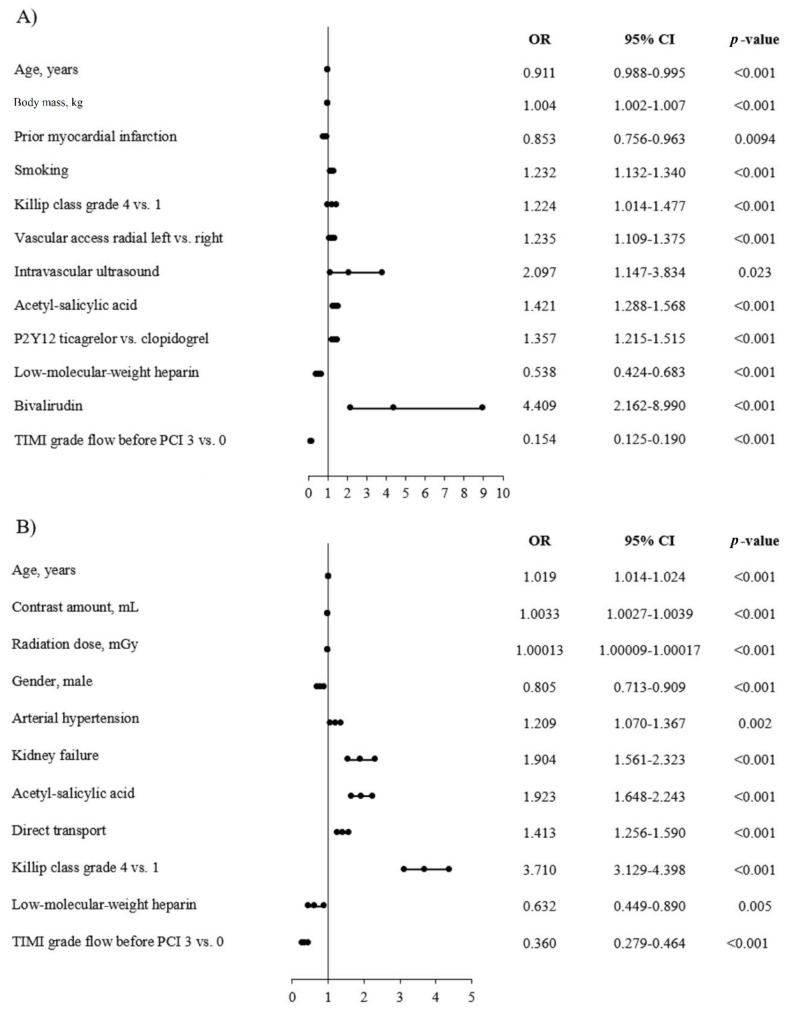
Predictors of TA and no-reflow phenomenon in the STEMI subgroup. (**A**) Thrombus aspiration. (**B**) No-reflow.

The results of univariate analysis are presented in Table 5. Among predictors of increased rate of AT usage, we found greater body mass, smoking, higher grade of Killip class at admission, type of vascular access, the use of intravascular ultrasound (IVUS), treatment with ASA before PCI and with new P2Y_12_ inhibitors (ticagrelor vs. clopidogrel), and treatment with bivalirudin. While among predictors of lower frequency of AT usage, we noted older age, prior MI, treatment with LMWH before PCI, and patency of culprit artery before PCI. Among predictors of higher NRP frequency: we demonstrated older age, contrast amount, radiation dose, arterial hypertension, kidney failure, treatment with ASA before PCI, direct transport to CathLab before PCI, and higher grade of Killip class at admission. Whereas, among predictors of lower NRP frequency, there were male gender, treatment with LMWH before PCI, and patency of culprit artery before PCI. The results of multivariate analysis are presented in Figure 3A,B.

### 3.6. Predictors of No-Reflow and AT in the NSTEMI Group

**Figure 4:**
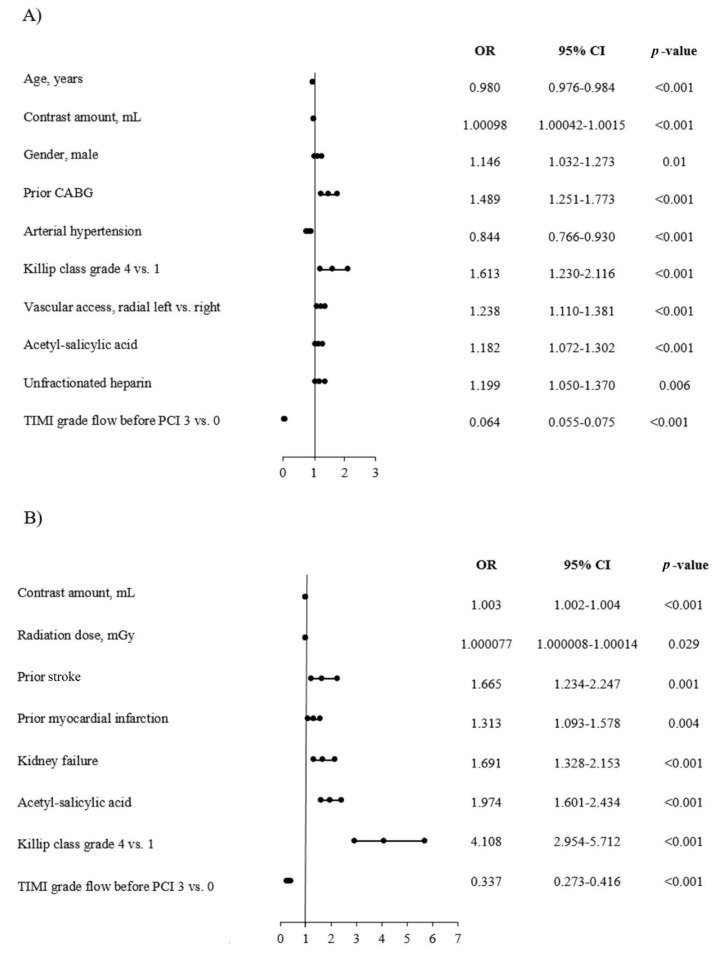
Predictors of TA and no-reflow phenomenon in the NSTEMI subgroup. (**A**) Thrombus aspiration. (**B**) No-reflow.

The results of the univariate analysis are presented in Table 6. Among the predictors of an increased TA usage rate, we found contrast amount, male gender, prior CABG, higher grade of Killip class at admission, type of vascular access, treatment with ASA, and unfractionated heparin. While among the predictors of lower TA frequency, we noted older age, arterial hypertension, and patency of culprit artery before PCI. Among predictors of higher NRP application frequency, we demonstrated greater contrast amount, radiation dose, prior stroke or MI, kidney failure, treatment with ASA, and higher grade of Killip class at admission. Whereas, among predictors of lower NRP frequency, there was patency of culprit artery before PCI. The results of the multivariate analysis are presented in Figure 4A,B.

## 4. Discussion

In the current study, we confirmed that the rate of TA decreased in the following years among the AMI group, as well as in the STEMI and NSTEMI subgroups of patients. This was accompanied by a significant increase in the percentage of pPCI without TA in all of the three selected groups of patients and was related in both cases to the change in treatment guidelines. The rate of NRP was significantly greater in the thrombectomy group compared to non-thrombectomy in the AMI group. The frequency of periprocedural strokes was significantly greater in the thrombectomy group compared to non-thrombectomy for AMI and NSTEMI patients. Among significant predictors of TA, younger patients, those in a more severe state at admission assessed by the Killip-Kimball scale, treated with ASA, and with occluded target coronary artery at baseline expressed by the TIMI grade scale were confirmed in the AMI group. TA use in patients from the NSTEMI group was significantly related to the contrast amount, prior CABG, male gender, arterial hypertension, and unfractionated heparin, whereas these factors were not significant for the AMI and STEMI groups. TA was significantly more often used in the AMI and STEMI groups of patients treated with the third generation of P2Y_12_ inhibitors compared to older patients, smokers, with COPD, those treated with bivalirudin, non-diabetics, and with greater body mass.

When considering predictors of NRP, among predictors of more frequent NRP occurrence in the NSTEMI group, there were prior stroke and MI; however, they were no longer significant predictors in the AMI and STEMI groups. On the other hand, older age, male gender, and arterial hypertension were predictors for AMI and STEMI but not among NSTEMI patients. Direct transport to the catheterization laboratory was a significant predictor of greater NRP occurrence, but only in the STEMI group, similarly to no use of LMWH. As common predictors of NRP occurrence in all of the three assessed groups of patients, we found contrast amount, radiation exposure, kidney failure, more severe clinical state at admission to hospital assessed by the Killip-Kimball scale grade, lack of patency of target coronary artery assessed by TIMI flow grade, and treatment with ASA.

As a consequence of the high frequency of suboptimal myocardial reperfusion after pPCI, the development of devices that evacuate coronary thrombus was observed. Additionally, pre-hospital (pre-H) administration of pharmacological therapy was also of interest. In clinical trials focusing on manual TA in pPCI, significant improvement in myocardial reperfusion has been shown. Whereas, there was no reduction in hard clinical endpoints [4,5]. In the majority of clinical studies, STEMI patients were assessed. However, in NSTEMI, 50% to 70% of all patients also display relevant thrombus burden in the culprit vessel [12]. The rates of angiographic NRP ranging from 15% to 40%, and depending on thrombus burden often enable suboptimal reperfusion [13]. Therefore, thrombectomy in patients with NSTEMI may be considered a useful intervention. Despite advances in STEMI treatment, the mortality rate remains considerably high. The most common mechanical strategies used in the setting of pPCI include coronary stenting, direct stenting, mesh-covered stents, self-expanding stents, deferred stenting, thrombectomy, distal protection devices, intra-aortic balloon pumping, left ventricular assist devices, and ischaemic conditioning [14]. Among various mechanisms suggested as an explanation for microvascular obstruction and NRP following pPCI, distal embolisation of thrombotic and/or atheromatous debris is believed to play an important role [15]. Langbag et al. showed that distal embolisation occurred in 11% of patients with STEMI treated with conventional pPCI and that its occurrence increased the risk of heart failure (HF) [16]. Direct stenting may be advantageous over stenting after predilatation in several aspects [17]. The MGuard stent was also found to be useful in preventing distal embolisation [18]. NRP is an independent predictor of morbidity and mortality among patients with STEMIs [19]. In patients who underwent pPCI for STEMI, an increased index of microvascular resistance (IMR) has independent predictive value for microvascular obstruction detection and further risk stratification of patients [20]. Thrombus burden evaluation could be helpful when individualising subjects who can benefit from TA in high-risk patients [21].

Tung et al. investigated the real-world outcomes of TA selective use during pPCI. With this strategy based on the physician’s judgment, no significant differences were detected between the TA and PCI-alone groups in terms of all-cause mortality or stroke during hospitalisation or at 30 days and 1 year of follow-up. In the subgroups of patients treated by physicians with a high pPCI volume, TA resulted in a better post-discharge survival rate at 1 year. In the current analysis, we were not able to assess this relationship due to a lack of follow-up. The British Cardiovascular Intervention Society study showed similar stroke rates in patients with and without thrombectomy during hospitalisation [22]. Granted stroke was a pre-specified endpoint in the TOTAL study; however, the results could be misleading. It has been proposed that stroke associated with TA may result from dislodged aspirated thrombus debris from the tip of the aspiration catheter during withdrawal or dislodgement of atheroma from the aorta caused by manipulation of the guiding catheter [23]. However, a significant increase in the risk of stroke was not detected in the thrombectomy group immediately after PCI (<12 h) but at 48 h, and there was also a trend of higher stroke risk in the thrombectomy group from 90 days to 1 year [24]. This could be caused by other unmeasured confounders, such as atrial fibrillation [25]. Another meta-analysis revealed that TA in pPCI was associated with a non-significant increase in the risk of stroke [26]. Kilic et al. demonstrated that TA was not associated with the occurrence of stroke at 30 days, supporting the possibility that the excess risk of stroke could not be fully attributed to TA [27]. However, this result was attributed largely due to the TOTAL study. Ghotak et al., in their meta-analysis involving 21,281 patients from 20 RCTs, showed no differences in mortality, recurrent MI, target-vessel revascularization, early or late ST, or net clinical benefits between TA and pPCI groups at the short- or long-term follow-up. Their results strongly suggest an increased risk of associated stroke with the use of manual TA as compared to those undergoing PCI alone during STEMI intervention [28]. Their data support the findings on increased stroke in the TOTAL trial and observations in prior meta-analyses [4,24]. The aetiology of these strokes is mostly embolic. For example, in the TAPAS trial, visible atherothrombotic material could not be retrieved out of the aspiration catheter in nearly 27% of patients [3]. It seems that TA is only beneficial in patients with moderate to high thrombus burden. On the other hand, if the mechanism of stroke is embolisation of thrombus from the coronary artery to systemic circulation, it is logical that the risk would be higher in patients with high thrombus burden. In patients with high thrombus burden, the increase in stroke could counterbalance an early benefit so that the effect on all-cause mortality at 1 year is neutral [29].

### 4.1. Predictors of No-Reflow Phenomenon

At present, the exact mechanism of NRP remains unclear, but clinical and laboratory findings suggest that it is related to the embolism of the capillary bed, ischemic injury, vascular endothelial dysfunction, production of oxygen free radical, inflammatory reaction, stress response, calcium overload, and other factors, such as ischemic injury, reperfusion injury, swelling of myocardial cells compressing microvascular vessels, and individual susceptibility [30]. The incidence of NRP after routine PCI, measured by TIMI grade, is 1% to 5%, and the incidence of NRP in AMI patients is from 2.3% to 41% [2]. Similar results have been observed in the current analysis. Even with good TIMI grade, myocardial perfusion is less than effective in 15% to 40% of cases with TIMI myocardial perfusion grade (TMPG) at 0 to 1 [31]. It has been proven that the development of NRP increases the risk of poor clinical outcomes, including death, re-MI, reduced left ventricular ejection fraction, left ventricular remodelling, malignant ventricular arrhythmia, HF, and cardiac rupture [32]. Because of its adverse effects, accurate detection of NRP, including identification of predictors, is crucial. Although, in some studies, NRP risk factors have been reported, they still showed discrepancies [33]. Predictors of NRP include lesion complexity, systolic hypertension, and low mass, and arterial hypertension was confirmed in the current analysis. Fajar et al. revealed that initial TIMI flow ≤1 and high thrombus burden are the most impacted NRP risk factors. We were not able to assess the impact of lesion type or thrombus burden in the current analysis due to a lack of data. Additionally, age, male gender, family history of CAD, smoking, diabetes mellitus, hypertension, and Killip class were proven to be associated with the risk of NRP [34]. The majority of these were confirmed in our analysis. Larger studies tend to concentrate on the whole AMI population and do not distinguish patients with STEMI [2]. A retrospective study of NRP in patients with STEMI undergoing direct PCI, published by Yang et al., was projected to find a scoring system to evaluate the risk of NRP. The results showed that age ≥65 years, no use of angiotensin-converting enzyme inhibitors/angiotensin receptor blockers, collateral circulation <grade 2, thrombosis burden ≥4 points, diameter of target lesion ≥3.5 mm, TA, and blood glucose >8 mmol/L were independent factors related to NRP [35]. In the angiographic sub-study of the TOTAL trial, routine TA did not result in improvement in the final MBG or TIMI flow among patients undergoing pPCI for STEMI [36]. It is speculative whether the thrombectomy catheter itself is responsible for abrupt IRA closure. In the TOTAL trial, distal embolisation was relatively uncommon and only occurred in 10% of patients in the PCI-alone group, and was reduced to 7% in the TA group. Patients with low thrombus burden were also included in the TOTAL trial and this patient population may be less responsive to thrombectomy. The KAMIR (Korea Acute Myocardial Infarction Registry) data demonstrated that TA showed clinical benefits only in special subgroups [37]. It was demonstrated in that registry that TA was more frequently applied in patients with worse clinical presentation and greater thrombotic burden. Therefore, apart from the fact that there are usually no differences in clinical outcomes between the PCI-alone and thrombectomy group, TA could be responsible for balancing the rates of possible adverse events of those two groups in the follow-up period. Additionally, it could be concluded based on the current analysis that the more frequent the use of TA in STEMI patients, the more frequent direct transport and related NRP are.

### 4.2. Anitplatelet Therapy

It was observed that TA, when left to physicians’ discretion, used in high-risk patients, was associated with bailout use of glycoprotein IIb/IIIa inhibitors and TIMI major bleeding, with no impact on 30-day clinical outcomes. Conversely, pre-H ticagrelor treatment predicted lower 30-day rates of ST or new MI without TA interaction. TA was associated with an increase of the risk for major TIMI bleeding, whereas pre-H ticagrelor was not. Concomitant use of TA and GPI may explain the higher incidence of bleeding events in TA groups [27]. Although TA did not improve the clinical outcomes in all patients, TA for LAD occlusion and the use of GP IIb/IIIa inhibitors with TA showed improvement in 12-month major adverse cardiac events (MACEs). The finding that TA may reduce cardiovascular mortality but increase stroke or TIA in those treated with glycoprotein IIb/IIIa inhibitors should be interpreted cautiously. Glycoprotein IIb/IIIa inhibitor use is likely to be highly correlated with thrombus burden. Another issue is related to the manner of randomisation [29]. Furthermore, in the INFUSE-AMI (Intracoronary Abciximab Infusion and Aspiration Thrombectomy in Patients Undergoing Percutaneous Coronary Intervention for Anterior ST-Segment Elevation Myocardial Infarction) trial, IS reduction by manual TA was non-existent [38]. The authors also suggested that morphine use is associated with slower uptake, delayed onset of action, and diminished effects of oral antiplatelet agents [39]. Based on the results of this study, some predictors of TA use in STEMI patients, like third-generation P2Y_12_ inhibitors, are more often used at baseline in STEMI patients compared to NSTEMI due to the greater probability of pPCI before admission to hospital.

### 4.3. Timing of Thrombus Aspiration

Sim et al. reported that manual TA during pPCI did not improve clinical outcomes at 12 months, whereas manual TA was associated with a higher risk-adjusted MACE rate compared to PCI alone in patients with longer total ischemic times. Subgroup analysis suggests that the impact of TA may become clinically relevant with longer total ischemic time, forming a U-shaped relationship. TA was associated with lower 12-month mortality and MACE in patients undergoing reperfusion between 4 and 6 h after symptom onset [40]. Fajar et al. found that delayed reperfusion increased the risk of NRP [34]. It has been suggested that the effects of TA on procedural and clinical outcomes may be related to multiple factors during STEMI, such as total ischemic time, IRA, and thrombus burden. In the VAMPIRE (Vacuum Aspiration Thrombus Removal) trial, a prospective multi-centre randomised study, greater reperfusion and clinical benefits by TA were shown in the subgroup of late-presenting (6–24 h) patients, compared to those receiving early reperfusion [41]. Similarly, in a study among 299 patients with STEMI (≤12 h), it was observed that the beneficial effect on optimal myocardial reperfusion of TA was more pronounced in the subgroups with total ischemic time >3 h, indicating that TA limited the adverse effects of total ischemic time prolongation on myocardial reperfusion [42]. Interestingly, in an analysis from a recent meta-analysis of 25 randomised trials, it was found that the beneficial effect of TA over PCI was only higher for longer ischemic times, for both mortality and MACE [43]. With further passage of time, the thrombus becomes harder to retrieve with manual TA catheters, resulting in a U-shaped curve of adverse outcomes with longer ischemic duration [42]. In addition, TA within this time window may favour optimised PCI with better sizing of stents or less ST as a result of effective thrombus removal [44]. In the presented study, we found that direct transport was a predictor of NRP but only in the STEMI subgroup. This was probably related to a more severe clinical condition before admission in these patient subgroups.

### 4.4. Microvascular Circulation

Jang et al. demonstrated that higher Killip class, higher peak troponin-I level, presence of multivessel disease, distal embolisation, and delayed reperfusion time were associated with higher IMR values, while the mechanical therapeutic strategy had no significant association with its lower values. Considering pharmacological strategies, the preloading of third-generation P2Y_12_ inhibitors showed a significant association with lower IMR value. In their study, Khan et al. showed that microvascular injury was more reduced in the third-generation P2Y_12_ inhibitor group. Results published by Jang et al. support the claim that third-generation P2Y_12_ inhibitors are more effective in lowering the IMR value than clopidogrel in patients with STEMI [45].

### 4.5. Thrombus Type

It is also claimed that types of thrombus affect mortality during PCI. Some studies have focused on the macroscopic appearance of the aspirated material, subdivided into white (fibrin-rich) and red (erythrocyte-rich) thrombus. White thrombus was observed in patients with a small thrombus burden and short ischemic time. Red thrombus was found in late-presenting patients with a higher risk of distal embolisation and cardiac mortality [39]. In the majority of both STEMI and NSTEMI patients, the key pathophysiological substrate is rupture of vulnerable plaque with subsequent coronary thrombosis. While in STEMI, the thrombus is mostly fibrin rich, leading to total vessel occlusion. The thrombus in many patients with NSTEMI is predominantly platelet rich and unstable [9]. Apart from this fact, the majority of patients with NSTEMI demonstrate relevant thrombus burden or even occluded coronary arteries and thus may benefit from thrombectomy [12]. However, it may be associated with an increased periprocedural stroke risk as shown in the current analysis in the group of patients with NSTEMI. Factors that may have an influence on this include the type of thrombus mentioned above, the time of transport to the hospital, or the patient’s condition before PCI (e.g., a higher percentage of patients after cardiac arrest).

### 4.6. Limitations

Limitations of our study include its retrospective observational design, lack of core-laboratory adjudication, and limited follow-up at the catheterization laboratory. We were not able to estimate the impact of several anatomical, procedural, and histological aspects of the study endpoints due to a lack of data. One of the key elements that may determine a certain bias in the analysis and in the interpretation is the lack of the thrombus burden and its type in the analysed database. Additionally, the use of TA and diagnosis of NRP, as well as other periprocedural complications, were left to the operator’s discretion. Whereas, among undoubted advantages of the current study, we distinguish very large patient numbers, which confer great statistical power. As a registry, it naturally includes higher-risk patients who are frequently excluded from RCTs and represents real-world practice not disturbed by the artificial randomisation requirements of the designed trials. The results regarding the relationship between the contrast volume used during the PCI and the radiation dose with the occurrence of NRP and the use of TA should be interpreted with a distance, because we should rather talk about the opposite relationship, namely that the prolongation of the procedure due to NRP and the use of TA is a consequence of greater use of contrast and greater radiation exposure during the procedure.

## 5. Conclusions

The frequency of thrombus aspiration has been steadily declining in recent years, regardless of AMI type among patients treated with pPCI. This was accompanied by a significant increase in the percentage of pPCI without TA in all of the three selected groups of patients. The frequency of periprocedural strokes was significantly greater in the thrombectomy group compared to non-thrombectomy for AMI and NSTEMI patients but not for STEMI. Predictors of TA and NRP significantly differ according to the type of MI.

## Figures and Tables

**Table 1:** Post-hoc analysis between frequencies of percutaneous coronary interventions with aspiration thrombectomy (PCI-TA) at selected years.

Years		*p*-Value		
Level 1	Level 2	AMI	NSTEMI	STEMI
2015	2014	<0.001	<0.001	<0.001
2016	2014	<0.001	<0.001	<0.001
2016	2015	0.001	0.003	<0.001
2017	2014	<0.001	<0.001	<0.001
2017	2015	0.46	0.001	<0.001
2017	2016	0.019	0.83	0.015
2018	2014	<0.001	<0.001	<0.001
2018	2015	<0.001	<0.001	0.072
2018	2016	<0.001	0.001	<0.001
2018	2017	<0.001	0.002	<0.001

**Table 2:** The relationship between the type of myocardial infarction (MI) and direct transport in light of no-reflow phenomenon (NRP) occurrence.

Type of AMI	Direct Transport	NRP			*p*-Value
		Total	Present	Absent	
NSTEMI	Total	90,849	607	91,456	0.038
	Absent	85,191 (93.15)	552 (90.94)	84,643 (93.17)	0.03
	Present	6261 (6.85)	55 (9.06)	6206 (6.83)	0.035
STEMI	Total	105,362	1420	103,942	<0.001
	Absent	80,007 (75.94)	928 (65.35)	79,079 (76.08)	<0.001
	Present	24,863 (23.92)	492 (34.65)	25,355 (24.06)	<0.001

Data are presented as count (percentage).

**Table 3:** Procedure-related complications according to type of myocardial infarction (MI)—non-thrombectomy and thrombectomy groups.

Variable (%)	Non-Thrombectomy Group				Thrombectomy Group			
	Overall	NSTEMI	STEMI	*p*-Value	Overall	NSTEMI	STEMI	*p*-Value
	*N* = 200,991	*N* = 98,492	*N* = 102,499		*N* = 16,777	*N* = 2570	*N* = 14,207	
No-reflow	0.82	0.58	1.06	<0.001	2.75 *	3.07 *	2.69 *	0.27
Puncture-site bleeding	0.13	0.14	0.12	0.11	0.14	0.19	0.13	0.41
Cardiac arrest	1.11	0.62	1.59	<0.001	2.49 *	1.36 *	2.69 *	<0.001
Allergic reaction	0.1	0.06	0.13	<0.001	0.45 *	0.23 *	0.49 *	0.05
CAP	0.19	0.2	0.18	0.41	0.2	0.39 *	0.16	0.03
Stroke	0.01	0.02	0.02	0.49	0.03 *	0.16 *	0.01	0.001
Dissection	0.14	0.14	0.14	0.9	0.07 *	0.16	0.05 *	0.08
Stent implantation	90.25	89.07	91.4	<0.001	91.19	86.59 *	91.96	<0.001
Stent type				<0.001				0.0016
Drug-eluting stent	87.19	86.41	87.95		87.89	82.88 *	88.73	
Bare-metal stent	2.32	1.91	2.73		2.21	1.83	2.28	
Bioresorbable scaffold	0.69	0.69	0.69		1.04	1.83 *	0.91	
No stent used	9.8	10.98	8.63		8.85 *	13.47	8.08	

CAP: coronary artery perforation. * when *p* value is <0.05, comparison of corresponding patient subgroups in the thrombectomy and non-thrombectomy groups.

**Table 4:** Predictors of aspiration thrombectomy and no-reflow in patients with acute myocardial infarction (AMI)—univariate analysis.

Variable	No-Reflow			Aspiration Thrombectomy		
	OR	95% CI	*p*-Value	OR	95% CI	*p*-Value
Age, years	1.018	1.014–1.022	<0.001	0.976	0.975–0.977	<0.001
Weight, kg	0.999	0.996–1.001	0.53	1.004	1.004–1.006	<0.001
Contrast amount, mL	1.004	1.003–1.004	<0.001	1.0002	1.0001–1.0004	0.06
Radiation exposure, mGy	1.0002	1.0002–1.0003	<0.001	1.0001	1.0001–1.0001	<0.0001
Gender, male	0.786	0.720–0.859	<0.001	1.214	1.173–1.257	<0.001
Diabetes mellitus	1.303	1.183–1.436	<0.001	0.718	0.689–0.749	<0.001
Prior stroke	1.974	1.667–2.338	<0.001	0.866	0.793–0.946	0.001
Prior myocardial infarction				0.573	0.547–0.600	<0.001
Prior percutaneous coronary intervention	0.729	0.649–0.820	<0.001	0.555	0.530–0.581	<0.001
Prior CABG	1.266	1.039–1.543	0.018	0.574	0.519–0.636	<0.001
Smoking	1.131	1.027–1.244	0.011	1.565	1.513–1.618	<0.001
Arterial hypertension	1.26	1.147–1.384	<0.001	0.785	0.760–810	<0.001
Kidney failure	2.25	1.967–2.573	<0.001	0.594	0.546–0.646	<0.001
Chronic obstructive pulmonary disease	1.698	1.347–2.141	<0.001	0.754	0.663–0.858	<0.001
Killip-Kimball class						
-class 4 vs. 1	5.683	4.932–6.548	<0.001	1.955	1.803–2.119	<0.001
Cardiac arrest before admission	2.36	2.009–2.773	<0.001	1.877	1.753–2.010	<0.001
Vascular access						
Femoral vs. radial right	1.368	1.242–1.508	<0.001	1.093	1.053–1.134	<0.001
Coronary angiography						
MVD + LMCA vs. SVD	1.403	1.162–1.693	<0.001	0.52	0.474–0.570	<0.001
Fractional flow reserve				0.225	0.135–0.376	<0.001
Intravascular ultrasound				0.805	0.652–0.995	0.04
Rotablation				0.269	0.148–0.489	<0.001
Acetyl-salicylic acid	1.295	1.182–1.420	<0.001	1.123	1.084–1.162	<0.001
Unfractionated heparin	1.164	1.037–1.308	0.01	1.063	1.020–1.108	0.003
P2Y_12_ inhibitor						
Ticagrelor vs. clopidogrel	1.29	1.040–1.601	0.02	1.6	1.467–1.745	<0.001
Thrombolysis	1.505	0.778–2.911	0.47	1.96	1.562–2.460	<0.001
Glycoprotein IIB/IIIa inhibitor 1 vs. 0				4.544	4.325–4.775	<0.001
TIMI flow grade before PCI 3 vs. 0	0.265	0.232–0.302	<0.001	0.061	0.056–0.066	<0.001

CABG: coronary artery by-pass grafting; LMCA: left-main coronary artery; MVD: multi-vessel disease; PCI: percutaneous coronary intervention; SVD: single-vessel disease; TIMI: Thrombolysis in Myocardial Infarction.

**Table 5:** Predictors of aspiration thrombectomy and no-reflow in patients with STEMI—univariate analysis.

Variable	No-Reflow			Aspiration Thrombectomy		
	OR	95% CI	*p*-Value	OR	95% CI	*p*-Value
Age, years	1.028	1.023–1.032	<0.001	0.985	0.983–1.015	<0.001
Weight, kg				1.005	1.004–1.006	<0.001
Contrast amount, mL	1.004	1.003–1.004	<0.001			
Radiation exposure, mGy	1.0002	1.0002–1.0003	<0.001	1.0001	1.0001–1.0001	<0.001
Gender, male	0.703	0.633–0.781	<0.001	1.143	1.100–1.188	<0.001
Diabetes mellitus	1.483	1.316–1.672	<0.001	0.860	0.820–0.902	<0.001
Prior stroke	1.941	1.559–2.416	<0.001			
Prior myocardial infarction				0.703	0.666–0.741	<0.001
Prior percutaneous coronary intervention	0.725	0.621–0.847	<0.001	0.659	0.624–0.695	<0.001
Prior CABG	1.403	1.033–1.906	0.038	0.653	0.566–0.754	<0.001
Smoking				1.421	1.370–1.475	<0.001
Arterial hypertension	1.404	1.258–1.568	<0.001	0.943	0.910–0.978	0.001
Kidney failure	3.133	2.630–3.732	<0.001			
Chronic obstructive pulmonary disease	2.228	1.672–2.968	<0.001			
Hypothermia				1.675	1.223–2.295	0.002
Direct transport	1.686	1.510–1.882	<0.001	1.233	1.185–1.283	<0.001
Killip-Kimball class -class 4 vs. 1	4.948	4.217–5.807	<0.001	1.485	1.362–1.618	<0.001
Cardiac arrest before admission	2.114	1.772–2.521	<0.001	1.441	1.340–1.550	<0.001
Vascular access Femoral vs. radial right	1.270	1.132–1.425	<0.001	1.018	0.977–1.060	<0.001
Coronary angiography MVD + LMCA vs. SVD	1.534	1.214–1.938	<0.001	0.580	0.522–0.644	<0.001
Fractional flow reserve				0.236	0.126–0.444	<0.001
Rotablation				0.262	0.123–0.558	<0.001
Acetyl-salicylic acid	1.400	1.254–1.563	<0.001	1.207	1.161–1.255	<0.001
Unfractionated heparin	1.312	1.141–1.510	<0.001	1.150	1.099–1.203	<0.001
P2Y_12_ inhibitor Ticagrelor vs. clopidogrel	1.218	0.945–1.569	<0.001	1.391	1.262–1.532	<0.001
Low molecular weight heparin				0.861	0.782–0.947	0.001
Glycoprotein IIB/IIIa inhibitor 1 vs. 0	2.010	1.700–2.377	<0.001	3.283	3.109–3.467	<0.001
TIMI flow grade before PCI 3 vs. 0	0.254	0.205	0.316	0.108	0.098–0.118	<0.001

CABG: coronary artery by-pass grafting; LMCA: left-main coronary artery; MVD: multi-vessel disease; SVD: single-vessel disease; TIMI: Thrombolysis in Myocardial Infarction.

**Table 6:** Predictors of aspiration thrombectomy and no-reflow in patients with NSTEMI—univariate analysis.

Variable	No-Reflow			Aspiration Thrombectomy		
	OR	95% CI	*p*-Value	OR	95% CI	*p*-Value
Age, years				0.970	0.967–0.973	<0.001
Weight, kg				1.006	1.004–1.008	<0.001
Contrast amount, mL	1.004	1.003–1.004	<0.001	1.0017	1.0012–1.0021	<0.001
Radiation exposure, mGy	1.0002	1.0002–1.0003	<0.001	1.0001	1.0001–1.0002	<0.001
Gender, male				1.435	1.314–1.569	<0.001
Diabetes mellitus	1.283	1.085–1.516	0.003	0.765	0.695–0.841	<0.001
Prior stroke	2.314	1.771–3.023	<0.001	0.804	0.649–0.996	0.045
Prior myocardial infarction	1.515	1.288–1.782	<0.001	0.708	0.642–0.780	<0.001
Prior percutaneous coronary intervention				0.652	0.589–0.722	<0.001
Prior CABG	1.628	1.251–2.118	<0.001	1.276	1.101–1.480	0.001
Smoking	1.211	1.015–1.444	0.032	1.540	1.415–1.677	<0.001
Arterial hypertension	1.267	1.058–1.518	0.009	0.805	0.741–0.875	<0.001
Kidney failure	2.128	1.719–2.635	<0.001	0.600	0.501–717	<0.001
COPD						
Hypothermia				3.398	1.356–8.514	0.026
Direct transport	1.358	1.028–1.795	0.03	1.178	1.017–1.365	0.031
Killip-Kimball class - class 4 vs. 1	5.648	4.119–7.745	<0.001	1.854	1.436–2.394	<0.001
Cardiac arrest before admission	1.956	1.298–2.948	0.001	1.676	1.337–2.100	<0.001
Vascular access Femoral vs. radial right	1.459	1.220–1.744	<0.001	1.033	0.939–1.136	<0.001
Coronary angiography MVD + LMCA vs. SVD	1.484	1.079–2.042	0.015	0.684	0.559–0.837	<0.001
Fractional flow reserve				0.390	0.161–0.943	0.013
Acetyl-salicylic acid	1.180	1.0003–1.392	0.049	1.099	1.010–1.197	0.029
Unfractionated heparin				1.204	1.076–1.347	<0.001
P2Y_12_ inhibitor Ticagrelor vs. clopidogrel				1.491	1.193–1.865	0.005
Thrombolysis				7.005	4.106–11.950	<0.001
Glycoprotein IIB/IIIa inhibitor 1 vs. 0	3.768	2.820–5.033	<0.001	6.253	5.500–7.110	<0.001
TIMI flow grade before PCI 3 vs. 0	0.330	0.276–0.399	<0.001	0.058	0.050–0.067	<0.001

CABG; coronary artery by-pass grafting, LMCA; left main coronary artery, MVD; multi-vessel disease, PCI; percutaneous coronary intervention, SVD; single-vessel disease.
